# Oxygenation Fluctuations Associated with Severe Retinopathy of Prematurity: Insights from a Multimodal Deep Learning Approach

**DOI:** 10.1016/j.xops.2023.100417

**Published:** 2023-10-21

**Authors:** Wei-Chun Lin, Brian K. Jordan, Brian Scottoline, Susan R. Ostmo, Aaron S. Coyner, Praveer Singh, Jayashree Kalpathy-Cramer, Deniz Erdogmus, R.V. Paul Chan, Michael F. Chiang, J. Peter Campbell

**Affiliations:** 1 Department of Ophthalmology, Oregon Health & Science University, Portland, Oregon; 2 Department of Neonatology, Oregon Health and Science University, Portland, Oregon; 3 Department of Ophthalmology, University of Colorado (CU) School of Medicine, Denver, Colorado; 4 Department of Electrical and Computer Engineering, Northeastern University, Boston, Massachusetts; 5 Department of Ophthalmology and Visual Sciences, University of Illinois at Chicago, Chicago, Illinois; 6 National Eye Institute, National Institutes of Health, Bethesda, Maryland; 7 National Library of Medicine, National Institutes of Health, Bethesda, Maryland

**Keywords:** Retinopathy of prematurity, Deep learning, Multimodal model, Machine learning, Time series data

## Abstract

**Purpose:**

Retinopathy of prematurity (ROP) is one of the leading causes of blindness in children. Although the role of oxygen in the pathophysiology of ROP is well established, a precise understanding of the dynamic relationship between oxygen exposure ROP incidence and severity is lacking. The purpose of this study was to evaluate the correlation between time-dependent oxygen variables and the onset of ROP.

**Design:**

Retrospective cohort study.

**Participants:**

Two hundred thirty infants who were born at a single academic center and met the inclusion criteria were included. Infants are mainly born between January 2011 and October 2022.

**Methods:**

Patient data were extracted from electronic health records (EHRs), with sufficient time-dependent oxygen data. Clinical outcomes for ROP were recorded as none/mild or moderate/severe (defined as type II or worse). Mixed-effects linear models were used to compare the 2 groups in terms of dynamic oxygen variables, such as daily average and the coefficient of variation (COV) fraction of inspired oxygen (FiO_2_). Support vector machine (SVM) and long-short-term memory (LSTM)-based multimodal models were trained with fivefold cross-validation to predict which infants would develop moderate/severe ROP. Gestational age (GA), birth weight, and time-dependent oxygen variables were used to develop predictive models.

**Main Outcome Measures:**

Model cross-validation performance was evaluated by computing the mean area under the receiver operating characteristic (AUROC) curve, precision, recall, and F1 score.

**Results:**

We found that both daily average and COV of FiO_2_ were associated with more severe ROP (adjusted *P* < 0.001). With fivefold cross-validation, the multimodal LSTM models had higher performance than the best static models (SVM using GA and 3 average FiO_2_ features) and SVM models trained on GA alone (mean AUROC = 0.89 ± 0.04 vs. 0.86 ± 0.05 vs. 0.83 ± 0.04).

**Conclusions:**

The development of severe ROP might not only be influenced by oxygen exposure but also by its fluctuation, which provides direction for future study of pathophysiological factors associated with severe ROP development. Additionally, we demonstrated that multimodal neural networks can be a method to extract useful information from time-series data, which may be a valuable methodology for the investigation of other diseases using EHR data.

**Financial Disclosure(s):**

Proprietary or commercial disclosure may be found in the Footnotes and Disclosures at the end of this article.

Retinopathy of prematurity (ROP) is an eye disease that affects the retina of preterm babies. It is a leading cause of childhood blindness worldwide.^1^^,^^2^ Blindness can occur from abnormal growth and development of blood vessels that lead to fibrovascular retinal detachment. Although there have been improvements in neonatal care, ROP is still a challenge for pediatric health care and needs early detection and intervention to prevent vision loss.^3^ A comprehensive understanding of the risk factors and underlying mechanisms of ROP is crucial for developing effective screening strategies and treatment plans.

Our understanding of the relationship between oxygen exposure and the incidence and severity of ROP has evolved over time. Several risk factors related to oxygen have been identified, including the utilization of any supplemental oxygen, the highest oxygen concentration, the duration of oxygen administration, and prolonged mechanical ventilation.^1^^,^^4^, ^5^, ^6^, ^7^, ^8^, ^9^, ^10^ The optimal range of oxygen saturation (SpO_2_) remains controversial, despite numerous large randomized-controlled studies that have evaluated different target ranges.^11^, ^12^, ^13^, ^14^, ^15^ Some studies found that the incidence and severity of ROP can be decreased by adjusting the delivered oxygen level through monitoring of SpO_2_ targets and avoiding hyperoxemia,^16^, ^17^, ^18^, ^19^ although this is balanced against the risk of intermittent hypoxemia. Intermittent hypoxemia refers to repeated episodes of decreased levels of oxygen in the blood, which may increase both overall morbidity and ROP risk.^19^, ^20^, ^21^

Our lack of precise understanding regarding the relationship between oxygen exposure and ROP risk may be due to the complexity of the relationship between oxygen delivery and blood-oxygen content. Because of the presence of lung disease and impaired oxygen transport, the relationship between inhaled oxygen delivered, measured by the fraction of inspired oxygen (FiO_2_), and oxygen content in the blood, as most easily measured by blood SpO_2_, is nonlinear. Moreover, SpO_2_ is an imperfect measurement of the total oxygen concentration of the blood for an infant receiving supplemental oxygen because, when hemoglobin is fully saturated, the true partial pressure of oxygen in the arterial circulation may be much higher than in an infant with fully saturated hemoglobin without supplemental oxygen. The true partial pressure of oxygen in the blood is usually known only through blood gas measurement in an infant. Further complicating our understanding is that SpO_2_ can vary from moment to moment, and FiO_2_ may be adjusted accordingly, which in and of itself may be an ROP risk factor. For example, several studies have suggested that not only high oxygen exposure but also variability in oxygen exposure is a significant predictor of severe ROP.^22^^,^^23^ There is support from preclinical data as well in the oxygen-induced retinopathy rat model, where McColm et al^24^ found that a more severe phenotype could be induced by fluctuating exposure to high oxygen concentrations.

Advances in big data analytics and deep learning offer new methods to understand complex relationships in biological data. In particular, the integration of deep learning with electronic health record (EHR) data can not only offer insights into fundamental pathophysiological relationships between clinical variables but can also be utilized in clinical prediction models.^25^, ^26^, ^27^, ^28^, ^29^ Moreover, deep learning models are flexible and capable of integrating different modalities of data, such as tabular data, free-text data, images, and time-series data, into a single predictive model.^30^^,^^31^ The ability to process and utilize multimodal inputs enhances the model's performance and the accuracy of predictions.^32^ The purpose of this study is to evaluate our gap in knowledge regarding time-dependent variables in the EHR and ROP and to compare prediction models using these variables compared to standard static variables (birth weight and gestational age [GA]).

## Methods

### Setting

The present study was conducted as part of a multicenter cohort study on ROP and was performed at the Oregon Health & Science University (OHSU), a large academic medical center in Portland, Oregon. In 2006, OHSU implemented an institution-wide EHR to handle all practice management, clinical documentation, order entry, medication prescribing, and billing. This study adhered to the tenets of the Declaration of Helsinki and was approved by the Institutional Review Board at OHSU (institutional review board #21403).

### Dataset

The primary dataset consisted of infants whose parents or guardians provided informed consent to participate in the Imaging and Informatics in ROP study between January 2011 and October 2022. A small number of infants born prior to 2011 were also included if they met the other inclusion criteria for the study. Inclusion criteria for the study were as follows: (1) ≤ 32 0/7 weeks of GA and (2) complete oxygen administration (FiO_2_) and saturation (SpO_2_) data for a minimum of 25 of the first 30 days in the EHR. Patients were excluded if they had missing EHR data, were born after 32 0/7 weeks of GA, or were transferred to the OHSU after birth. The FiO_2_ and SpO_2_ data were extracted from the EHR data warehouse at OHSU. The majority of records (89%) had data intervals of 1 to 3 hours, while a few infants had more frequent recordings on specific days. Aggregated SpO_2_ variables, such as daily mean, maximum, minimum, and coefficient of variation (COV), were calculated from these raw data to mitigate the impact of variability in the frequency of oxygen records. Daily aggregation smoothes out short-term fluctuations and reduces noise, facilitating the identification of meaningful trends. The diagnostic procedure for each retinal examination comprised a clinical bedside evaluation of the retinae, coupled with an image-based ROP diagnosis performed by 3 experienced ROP graders (J.P.C., M.F.C., and S.O.). The image-based diagnosis relied on all 5 retinal fundus image views and was based on the International Classification of Retinopathy of Prematurity criteria. The first group consisted of infants who either did not exhibit ROP or had mild ROP, which did not require treatment. The second group was comprised of infants who exhibited moderate to severe ROP (type 2 or more severe) and may or may not have received treatment.

### Statistical Analysis

Continuous variables were expressed as mean and standard deviation, and a Welch's t-test was used to compare the difference between ROP severity groups. The distribution of categorical variables was described using frequency and percentage values, and group comparisons were performed using the chi-square tests. Mixed-effects linear models were used to compare the 2 groups in terms of the daily mean of SpO_2_, daily COV of SpO_2_, daily mean of inspired oxygen (FiO_2_), daily COV of FiO_2_, and the daily proportion of high oxygen exposure while accounting for the correlation among repeated measurements obtained from the same infant over time. A linear mixed model was initially fit for each variable with 5 predictors: GA, birth weight, day of life, ROP severity group, and an interaction term between the day of life and the ROP severity group. The significance of the interaction term was assessed, with a statistically significant result indicating that the relative effect of the ROP severity group changed over time, thereby making the generalization of group differences across time infeasible. For instances in which the interaction term was not significant, it was removed, and the model was refitted with GA, birth weight, the day of life, and the ROP severity group as the main predictors, thereby allowing for a longitudinal comparison between groups. Data analysis was conducted using the nlme package in R version 4.2.3 (R Foundation for Statistical Computing), with a 5% significance level applied for hypothesis testing.

### Machine Learning and Deep Learning Models

Due to the nature of collinearity among the oxygen exposure and saturation data, we compared 2 popular machine learning models, random forest and support vector machine (SVM), to explore the possible oxygen parameters related to severe ROP onset. We applied recursive feature selection to select the most relevant variables, including GA, average FiO_2_, the COV of FiO_2_, and the proportion of hyperoxemia. Next, we used GA to develop the first SVM model and used the 4 selected variables to develop the second SVM model. The fivefold cross-validation technique was employed to train models, and the outputs of each submodel were used to calculate average results. Cross-validation is a commonly used method to evaluate the performance of a predictive model on an independent dataset, which is a resampling procedure that iteratively trains and tests the model using different proportions of the dataset. The goal of cross-validation is to provide an estimation of the model's generalizability. A grid search was performed to optimize the hyperparameters of the models. These models were implemented using scikit-learn in the Python environment.

**Figure 1:**
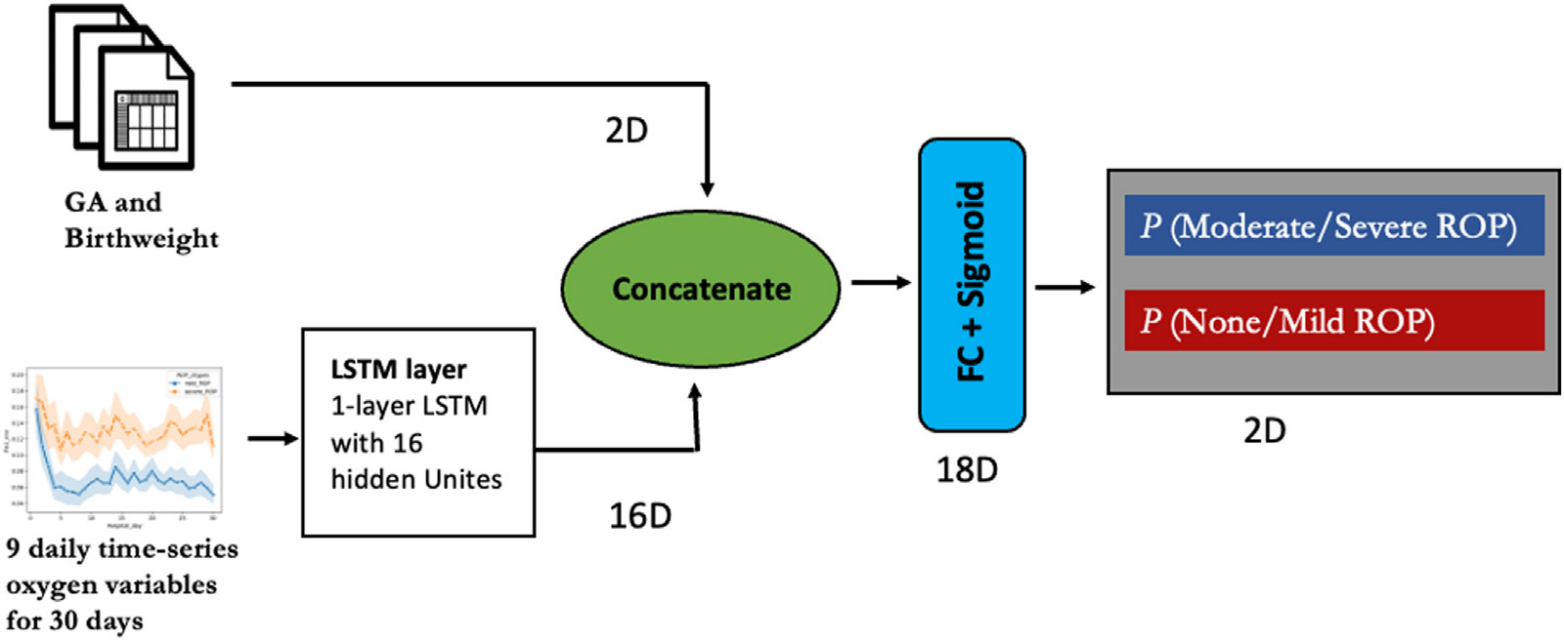
Architecture of multimodal long-short-term memory (LSTM) prediction model. Architecture for the multimodal-LSTM prediction model with 16 hidden units using oxygen time-series and static data. FC = fully connected layer; GA = gestational age; ROP = retinopathy of prematurity.

Lastly, we developed a multimodal deep learning model to extract information from time-series oxygen data and incorporate it with 2 static variables to improve the predictive model performance. The model was implemented using PyTorch in the Python environment. Figure 1 shows the model architectures of the multimodal neural networks. The long-short-term memory (LSTM) layers were used to capture the information from the 9 time-series oxygen data, including the daily proportion of normal FiO_2_ (≤ 0.23), daily mean, maximum, minimum, and COV of FiO_2_ and SpO_2_. The static data included GA and birth weight. The neural network model architectures (hidden layers and directions of LSTM) and hyperparameters (optimizers, learning rates, batch size, and dropout rates) were optimized. The optimized neural network model was trained with Adam optimizer and used ReLU as the activation function. The batch size was 4 with a learning rate of 0.001. The parameters of the last hidden layer (dimensions = 16) were contacted with static data and connected with one more dense layer and the final out layer with a sigmoid function. Model performance was evaluated by computing the mean area under the receiver operating characteristic (AUROC) curve, precision, recall, and F1 score.

## Results

### Dataset Characteristics

This study included 230 infants who were born at OHSU and met the inclusion criteria, of whom 37 did not develop ROP, 106 developed mild ROP, 39 developed type 2 ROP, and 48 developed treatment-requiring ROP (Table 1). The none/mild ROP subjects were grouped as mild or no ROP (n = 143), and moderate/severe ROP subjects were grouped as moderate or severe ROP (n = 87). Moderate to severe ROP was associated with lower birth weight (*P* ≤ 0.001) and lower GA at birth (*P* ≤ 0.001). No significant differences were observed for race, gender, and ethnicity. Birth weight and GA were taken into account in the adjusted statistical analyses.

**Table 1:** Baseline Demographic Characteristics

Characteristics	None/Mild ROP (n = 143)	Moderate/Severe ROP (n = 87)	*P* Value
	Mean (SD)	Mean (SD)	
Birth weight, gram	975.3 ± 241.8	716.2 ± 193.3	< 0.001∗
Gestational age, week	27.3 ± 1.5	25.4 ± 1.3	< 0.001∗
	N (%)	N (%)	
Sex			
Male	79 (55)	49 (56)	0.982
Female	64 (45)	38 (44)	0.982
Race			
White	110 (77)	67 (77)	1.0
Black	10 (7)	2 (2)	0.139
Asians	6 (4)	2 (2)	0.713
Others and unknown	17 (12)	16 (18)	0.18
Hispanic ethnicity	31 (22)	24 (28)	0.341

ROP = retinopathy of prematurity; SD = standard deviation.

Calculation based on Pearson chi-square test (sex, race, and Hispanic ethnicity) and Welch's t-test (birth weight and gestational age).

∗ *P* values were 2-sided, and *P* ≤ 0.05 was considered statistically significant.

### Trends in FiO_2_ and SpO_2_

**Figure 2:**
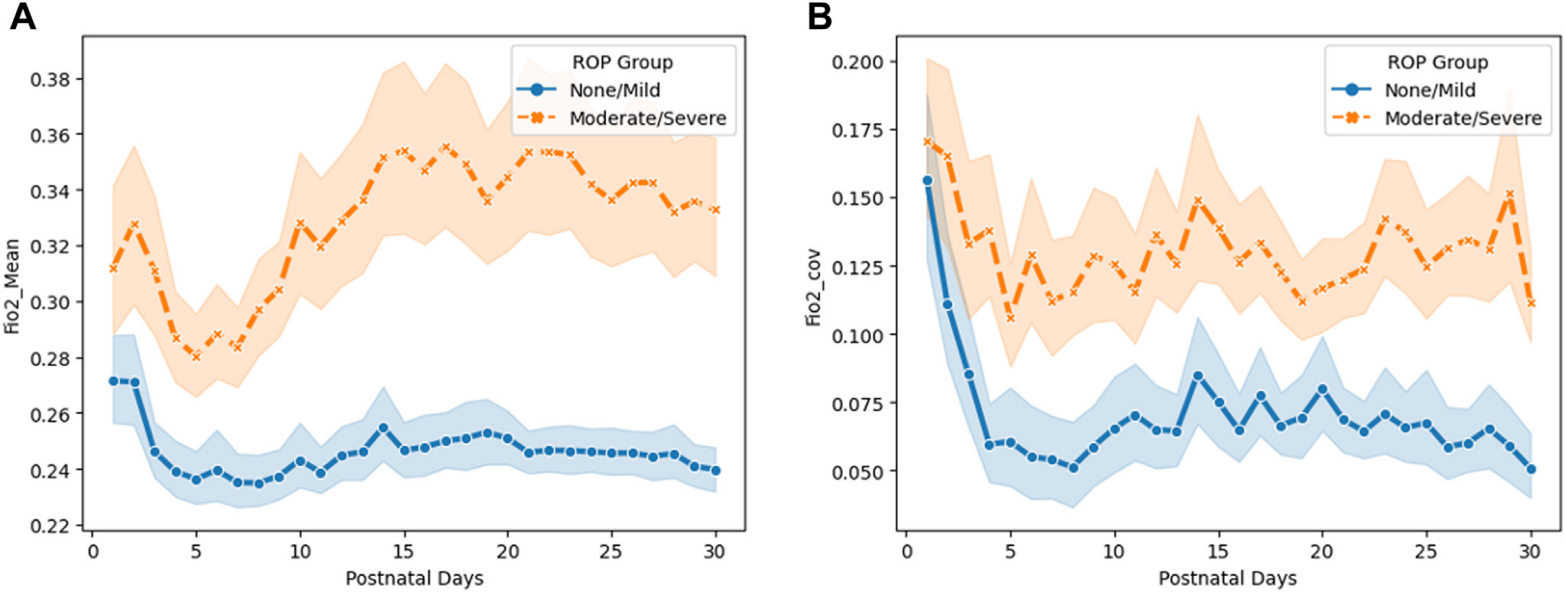
Descriptive characteristics of fraction of inspired oxygen (FiO_2_) during the first 1 postnatal month. **A,** Daily average FiO_2_ for 1 month. **B,** Daily average FiO_2_ coefficient of variation (COV) for 1 month. ROP = retinopathy of prematurity.

The trend of the average daily FiO_2_ for the first month is shown in Figure 2A, while Figure 2B displays the daily COV of FiO_2_ for the first month. The daily average FiO_2_ percentages were significantly higher for patients with moderate to severe ROP compared to those with no or mild ROP, as shown in Table 2 (adjusted *P* < 0.001). The average FiO_2_ percentage decreased for both groups during the first 5 days, then reached its peak at approximately 12 to 14 days, and slightly declined over the next 2 weeks for the group with severe ROP. The group with mild or no ROP displayed a relatively stable trend with FiO_2_ values remaining below 25%. Similarly, the daily COV of FiO_2_ was significantly higher for patients with severe ROP compared to those in the no or mild ROP group (adjusted *P* < 0.001). The daily COV of FiO_2_ declined during the first week and fluctuated during the subsequent days.

**Table 2:** Longitudinal Variables between Study Groups Using Linear Mixed-Effect Models

Variables	Adjusted Slope Coefficient (Standard error)	Adjusted *P* Value
Daily average FiO_2_	0.0385 (0.0070)	< 0.001∗
Daily FiO_2_ coefficient of variation	0.0382 (0.0056)	< 0.001∗
Proportion of daily FiO_2_ ≤ 0.23	−0.0667 (0.0338)	0.043∗
Daily average SpO_2_	−0.5704 (0.959)	0.1509
Daily SpO_2_ coefficient of variation	0.0032 (0.0058)	0.5801

FiO_2_ = fraction of inspired oxygen; SpO_2_ = oxygen saturation.

The adjusted slope coefficient demonstrates the average change in values between the 2 groups over time after controlling for birth weight and gestational age. The positive slope suggests that the none/mild retinopathy of prematurity group average value is less, and a negative slope indicates a greater average value for the none/mild retinopathy of prematurity group.

∗ *P* values were two-sided, and *P* ≤ .05 was considered statistically significant.

**Figure 3:**
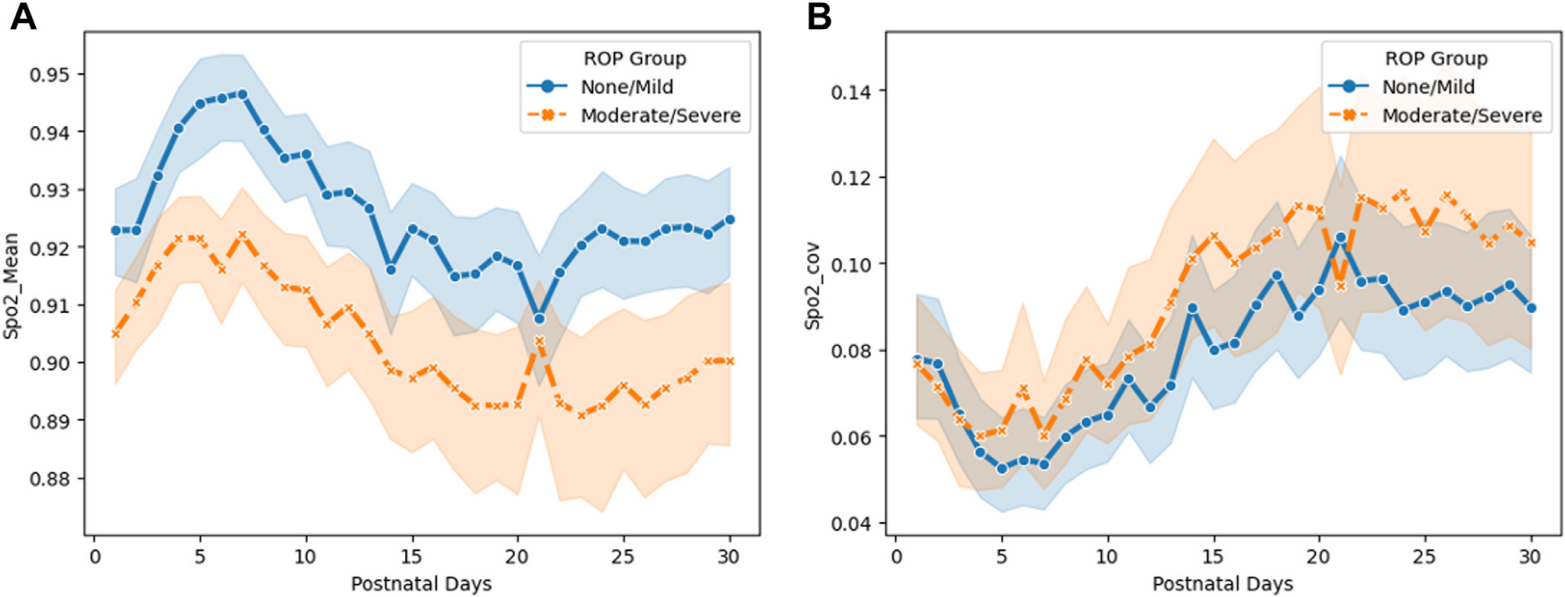
Descriptive characteristics of oxygen saturation (SpO_2_) during the first 1 postnatal month. **A,** Daily average SpO_2_ for 1 month. **B,** Daily average SpO_2_ coefficient of variation (COV) for 1 month. ROP = retinopathy of prematurity.

Figure 3A shows the trends of average daily SpO_2_, and Figure 3B displays the daily COV of SpO_2_ for the first month. The daily average SpO_2_ percentage was slightly higher but without statistical significance for patients with moderate or severe ROP (adjusted *P* = 0.151), as shown in Table 2. The average SpO_2_ increased in the first 5 days and then declined until day 20; afterward, it remained steady or slightly increased. In contrast, there was no significant difference between the daily COV of SpO_2_ between the 2 groups (adjusted *P* = 0.58). The daily COV of SpO_2_ decreased in the first 5 days, then rose until day 20. After that, the daily COV of SpO_2_ remained stable. Furthermore, in Table 2, we found that patients with severe ROP had a lower proportion of time with ambient air FiO_2_ compared to the subjects in the no or mild ROP group (adjusted *P* = 0.043).

### Predictive Model Performance

**Figure 4:**
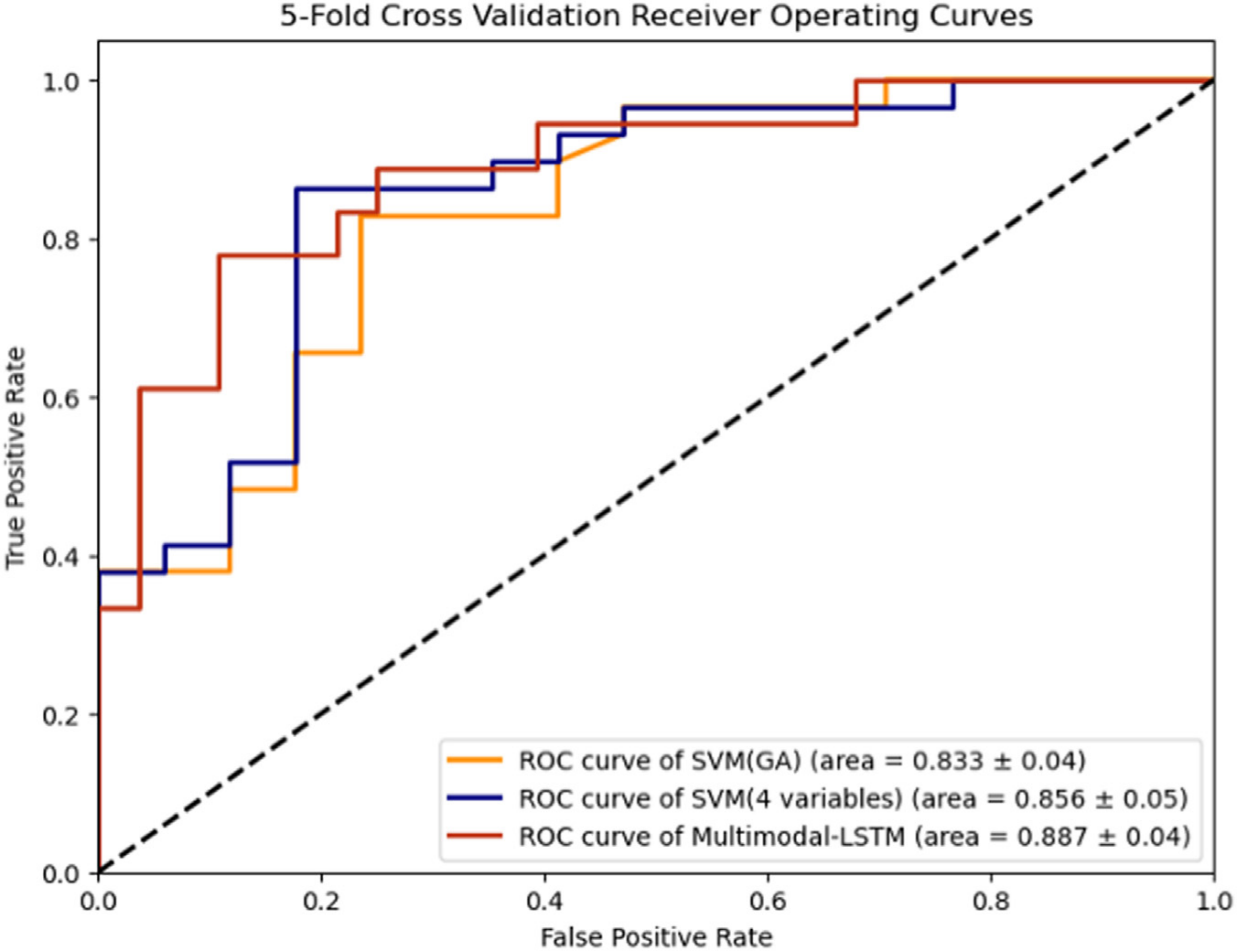
Receiver operating characteristic (ROC) curves for predictive models. ROC curves are shown for the 3 predictive models using different input data. 4 variables = 4 variables, including gestational age, daily average fraction of inspired oxygen (FiO_2_), daily FiO_2_ coefficient of variation, and proportion of daily FiO_2_ ≤ 0.23; GA = gestational age; LSTM = long-short-term memory; SVM = support vector machine.

The performance of the SVM models and multimodal neural networks for predicting severe ROP is shown in Table 3. The results of the fivefold cross-validation showed that the performance of the multimodal neural networks had the highest AUROC and F1 score, followed by the SVM model using GA and the average of 3 FiO_2_ features, and SVM models trained solely on GA. The mean AUROC of the multimodal neural networks was 0.89 ± 0.04 compared to 0.86 ± 0.05 for the SVM with 4 variables and 0.83 ± 0.04 for the SVM with GA alone (Figure 4). Additionally, the multimodal neural networks demonstrated the highest F1 score at 0.85 ± 0.06, followed by the SVM with 4 variables at 0.82 ± 0.07 and the SVM with GA alone at 0.80 ± 0.06.

**Table 3:** Performance Metrics for Prediction Models

	Surgical Outcomes Predictions				
	Model Inputs	AUROC	Precision	Recall	F1 Score
SVM-GA	*GA*	0.83	0.82	0.79	0.80
SVM-GA-FiO_2_	*GA + FiO*_*2*_*∗*	0.86	0.82	0.82	0.82
**MNN-LSTM**	***T + S***	**0.89**	**0.87**	**0.84**	**0.85**

AUROC = area under the receiver operating characteristic curve; FiO_2_ = fraction of inspired oxygen; FiO_2_∗, 3 variables including daily average FiO_2_, daily FiO_2_ coefficient of variation, and proportion of daily FiO_2_ ≤ 0.23; GA = gestational age; LSTM = long-short-term memory; MNN = multimodal neural network; *S* = static data (gestational age and birth weight); SVM = support vector machine; *T* = time-series oxygen variables for the first postnatal month.

The bold in the table is maximum values of that evaluation metrics.

## Discussion

In this study, we developed statistical, machine learning, and multimodal deep learning models to investigate the correlations between several quantitative oxygen parameters recorded in the EHR and the onset of severe ROP. The 2 key findings of our study were (1) that a significant positive correlation exists between both daily average FiO_2_ and variability of FiO_2_ with severe ROP, even after adjusting for birth weight and GA, and (2) that a multimodal deep learning model can capture richer information from time-series EHR data and provide better prediction results. These findings align with previous studies showing associations between variability in oxygen requirements being associated with outcomes such as bronchopulmonary dysplasia and overall mortality in neonates.^14^^,^^33^^,^^34^

The pathophysiological mechanism behind the relationship between oxygen fluctuation and end organ damage, such as severe ROP, is still not fully understood. Perelli et al^35^ discussed one potential mechanism, demonstrating in a mouse model that transient neonatal exposure to excess oxygen disrupts the formation of the angiogenic astrocyte and suppresses astrocyte proliferation resulting in a disturbance of their spatial patterning. This disturbance caused vascular defects similar to those seen in infants with ROP. This study suggests that fluctuations in oxygen exposure can disrupt the molecular pathways that control astrocyte proliferation, causing an increase in astrocyte numbers and interfering with retinal angiogenesis, ultimately increasing the risk of developing severe ROP. Furthermore, other studies have suggested that fluctuating oxygen exposure may lead to oxidative stress, which might also increase the risk of severe ROP.^36^^,^^37^

However, achieving the goal of titrating FiO_2_ to specific SpO_2_ targets and limiting fluctuations in FiO_2_ and SpO_2_ remains challenging because of the large number of factors that affect SpO_2_ and how quickly the infants’ clinical state can change.^38^ As such, improving the FiO_2_–SpO_2_ relationship remains a persistent challenge in neonatal care. Recent efforts have been made to develop methods for reducing FiO_2_ and SpO_2_ variations, including automated control systems^39^ and feedback systems.^40^ Nevertheless, further research is still needed to understand the pathobiology of oxygen fluctuations better and to aid in developing effective strategies for minimizing their effects. Our study contributes to this effort by highlighting the importance of monitoring oxygen exposure and, to the extent that it can be achieved, reducing unnecessary fluctuations in iatrogenic oxygen exposure to prevent severe ROP. In the future, further studies will be needed to explore the specific mechanisms related to iatrogenic oxygen fluctuations and the onset of severe ROP.

This study further demonstrated that multimodal neural networks could improve model performance by integrating different sources of input data. The model developed in this study incorporates 2 types of information, including static data (GA and birth weight) and time-series oxygen data. We used the LSTM layers to process and extract useful information from time-series EHR data. Long-short-term memory is a subtype of the recurrent neural network, which can retain long-term information and selectively forget irrelevant information, making it well-suited for extracting information from time-series EHR data.^41^ Therefore, LSTM layers can identify temporal patterns and dependencies in the data, allowing the model to make more informed predictions compared to models that use static or aggregation data alone. In our study, the multimodal model demonstrated the highest AUROC at 0.89 ± 0.04, outperforming the SVM models with 4 variables and SVM models trained solely on GA. Our results suggest that utilizing the multimodal deep learning model could be a valuable method for developing a prediction model for the onset of severe ROP. This approach might be useful for other oxygen-related diseases.

This study has several limitations. First, our study was conducted in a single academic center, which could limit the generalizability of the results to other populations. To address this limitation, we intend to validate the models using a similar dataset from an external institute in the future. Second, the sample size of infants in this study was limited to those infants born at the single institution. We excluded infants transferred to OHSU to maintain the accuracy and completeness of oxygen data during the crucial first 30 days after birth. The time period between each oxygen data measurement was not standardized in this study; thus, we used the COV to represent the daily oxygen fluctuation. In future studies, it would be ideal to use a fixed time stamp for oxygen data to overcome this limitation. Additionally, while birth weight and GA were adjusted for, illness severity was not included in the model. Furthermore, it is important to note that the feasibility of obtaining similar oxygen measurements in smaller hospitals with limited resources may pose unique challenges, but the use of daily aggregation and technological solutions, such as EHR integration or monitoring devices with cloud uploading, may help address potential resource gaps.

In conclusion, abundant time-series oxygen data from EHR can be easily extracted and utilized in predictive modeling for the onset of severe ROP. Our study indicates that not only oxygen exposure but also its fluctuation may play a role in the development of severe ROP, providing direction for future study of pathophysiological factors associated with oxygen exposure and fluctuation in severe ROP development. Additionally, we demonstrated that multimodal neural networks could be a method to extract useful information from time-series data, which may be a valuable methodology for the investigation of other diseases using EHR data.

## Declaration of Generative AI and AI-Assisted Technologies in the Writing Process

During the preparation of this work, the author used Chat-GPT in order to improve readability and language. After using this tool, the authors reviewed and edited the content to ensure all statements were correct. The authors take full responsibility for the content of the publication.
